# Results of a national survey of substance use treatment services for youth under community supervision

**DOI:** 10.1186/s40352-023-00233-w

**Published:** 2023-07-29

**Authors:** Danica K. Knight, Rod R. Funk, Steven Belenko, Michael Dennis, Amanda L. Wiese, John P. Bartkowski, Richard Dembo, Katherine S. Elkington, Patrick M. Flynn, Philip W. Harris, Aaron Hogue, Lawrence A. Palinkas, Angela A. Robertson, Christy K. Scott

**Affiliations:** 1 Institute of Behavioral Research, Texas Christian University, 3034 Sandage Avenue, Fort Worth, TX 76129 USA; 2 Chestnut Health Systems, 1003 Martin Luther King Jr. Drive, Bloomington, IL 61701 USA; 3 Temple University, 1801 N. Broad Street, Philadelphia, PA 19122 USA; 4 Chestnut Health Systems, 448 Wylie Drive, Normal, IL 61761 USA; 5 University of Texas at San Antonio, 1 UTSA Circle, San Antonio, TX 78248 USA; 6 University of Southern Florida, 4202 E. Fowler Avenue, Tampa, FL 33620 USA; 7 Columbia University and New York State Psychiatric Institute, 1051 Riverside Drive, New York, NY 10032 USA; 8 Partnership to End Addiction, Family and Adolescent Clinical Technology & Science (FACTS), 485 Lexington Avenue, 3rd Floor, New York, NY 10017 USA; 9 University of Southern California, 669 W. 34th Street, Los Angeles, CA 90089 USA; 10 Mississippi State University, 1 Research Blvd., Suite 103, Starkville, MS 39759 USA; 11 Lighthouse Institute, Chestnut Health Systems, 221 W. Walton, Chicago, IL 60610 USA

**Keywords:** Juvenile justice, Substance use, Drug treatment, Behavioral health, Inter-agency collaboration

## Abstract

**Background:**

Despite the heightened risk for substance use (SU) among youth in the juvenile justice system, many do not receive the treatment that they need.

**Objectives:**

The purpose of this study is to examine the extent to which youth under community supervision by juvenile justice agencies receive community-based SU services and the factors associated with access to such services.

**Methods:**

Data are from a nationally representative sample of Community Supervision (CS) agencies and their primary behavioral health (BH) partners. Surveys were completed by 192 CS and 271 BH agencies.

**Results:**

SU services are more often available through BH than CS for all treatment modalities. EBPs are more likely to be used by BH than by CS. Co-location of services occurs most often in communities with fewer treatment options and is associated with higher interagency collaboration. Youth are more likely to receive services in communities with higher EBP use, which mediates the relationship between the availability of SU treatment modalities and the proportion of youth served.

**Conclusion:**

Findings identify opportunities to strengthen community systems and improve linkage to care.

## Background

Well-established co-morbidity between delinquency and substance use (SU) is a serious correctional and public health concern. Because almost 50% of SU disorders (SUDs) begin by age 20 (R. C. Kessler et al., 2005), adolescence is a key period for prevention and intervention. SU treatment in adolescence can offset trajectories of SU/SUD in adulthood and decrease related negative outcomes, including recidivism and violence (Cuellar et al., 2004; Henggeler & Sheidow, 2012; Hoeve et al., 2013). Although SU treatment needs among juvenile justice (JJ) youth are greater than youth in the general population (Chassin, 2008; International & America, 2004), only a third of JJ youth with SUDs received treatment in the previous year (SAMHSA, 2013; Wasserman et al., 2021). Indeed, many JJ youth who need treatment do not receive it (Dennis et al., 2019; Johnson et al., 2004) even when in secure residential facilities (Mulvey et al., 2007). The aims of this paper are to (a) document gaps in availability of SU treatment nationally for this population and (b) identify opportunities to strengthen and expand the existing service infrastructure.

### Substance use service needs and linkage to treatment

Most JJ youth are under formal community supervision (CS) by a juvenile justice agency (Hockenberry & Puzzanchera, 2018). CS includes court supervision, probation, and parole (Champion, 1992). Typically, upon initial JJ system entry, an intake officer reviews charges, interviews the youth and parent to obtain a social history, and administers screening instruments (e.g., risk and needs) (Bowser et al., 2018). This information informs court decisions. For youth with identified SU problems, there are generally two routes to behavioral health (BH) services: a judicial determination following adjudication (e.g., conditions of supervision) or diversion at court intake. In either case, referral to treatment is the responsibility of probation personnel. When SU treatment is provided by an independent community-based provider, coordination between the CS agency and the BH agency is necessary before clinical assessment and treatment can occur. At this juncture, cases can be misplaced or overlooked (Belenko et al., 2017).

The Juvenile Justice BH Services Cascade (Cascade) provides a framework for tracking and measuring unmet treatment needs and for guiding efforts to improve access to and participation in community-based SU treatment (Belenko et al., 2017). Based on the HIV care cascade (Mugavero et al., 2013), it provides a structure and visual representation of the ideal sequence of steps through which cases travel, from preliminary screening to engagement in treatment. The Cascade comprises six distinct interrelated activities that are essential for identifying SU problems and moving youth into appropriate clinical services: screening and assessment, identification of need, referral, initiation, engagement, and continuing care (Knight et al., 2016). Implicit in the Cascade is that as BH services are added to JJ orders, communication and coordination are required. Preliminary evidence suggests two points in the Cascade where the percentage of youth retained drops significantly: at the transitions from need identification to referral (63% not referred) and upon initiation to engagement (49% do not engage for a minimum of 6 weeks) (Dennis et al., 2019). Significant system-level barriers to referral and treatment initiation in the community include a lack of available treatment options (Ahrnsbrak et al., 2017; Bose et al., 2017), large probation caseloads, high stress among staff (Wasserman et al., 2021), and minimal communication and collaboration between JJ and BH agencies (Bowser et al., 2018; Elkington et al., 2020; Taxman & Belenko, 2011; Welsh et al., 2021).

### Effectiveness and availability of adolescent SU treatment

Various SU treatment intervention programs and practices are available for adolescents (Bender et al., 2010; Hogue et al., 2018; NIDA, 2014; Sexton and Alexander, 2000; Tanner-Smith et al., 2016; Welsh et al., 2021). Cognitive-behavioral therapy (CBT) and family-based approaches are among the most effective for reducing SU among legally and non-legally involved youth (Chorpita et al., 2011; Liddle, 2004; Perrino et al., 2000; Tripodi et al., 2011; Webb et al., 2002). However, it is widely believed that standard treatment quality for adolescent SU is mediocre-to-inadequate due to a host of factors headlined by the absence or modest quality of evidence-based practices (EBPs), insufficient provider training, and little quality monitoring (Brewer et al., 2017; Hogue et al., 2018; McLellan & Meyers, 2004). EBPs for SU treatment are not routinely used with fidelity in clinical practice (Chorpita et al., 2011) and 90% of publicly-funded mental health and JJ systems do not use them in SU treatment (Hoagwood & Olin, 2002). Youth on CS have low rates of service use (Teplin et al., 2005), and the vast majority lack access to EBPs (Young et al., 2007). Even when CS systems make referrals to treatment, few youth follow up (Teplin et al., 2005), and receipt of EBPs for SU is unlikely (Scott et al., 2019). Another challenge to the use of EBPs is their cost, often putting them beyond the reach of community-based BH service providers (Englund et al., 2008).

Much of what is known about access to SU treatment among JJ youth comes from studies of youth who are incarcerated, even though 75% of justice-involved youth are supervised in the community (Hockenberry & Puzzanchera, 2019). An early national survey found that 36.7% of secure juvenile correctional facilities provided SU treatment (Marsden & Straw, 2000). A later national survey of 72 counties featuring 165 juvenile residential facilities (i.e., local detention centers/jails, community correctional programs, and residential facilities) found that 75% offered drug and alcohol education (a non-intensive intervention without strong empirical support), 40% offered brief group counseling (1–4 h a week; only 14% of CS youth attended), 21% offered intensive outpatient services (5–25 h per week; less than 1% of JJ youth attended), and family-based services were scarce (Young et al., 2007). National data indicate that community programs for JJ youth were more likely to employ staff qualified to deliver SU treatment, involve families in treatment, and assess treatment outcomes (Henderson et al., 2007). However, institutional programs were more likely to provide comprehensive medical, mental health, SU, and case management services. These findings are consistent with studies of quality gaps in adolescent SU treatment programs in general (Brannigan et al., 2004; Knudsen, 2009) and highlight the need to better quantify and improve service quality for JJ youth.

### Service system factors

Reforms aimed at reducing the number of youth in long-term secure facilities have resulted in more youth being supervised within their communities (Fabelo et al., 2015). Accordingly, juvenile and family courts must rely on community-based SU treatment providers for services. Although many JJ systems intend to identify and link youth to needed services, their efforts are hampered by an underestimation of SU problems faced by probation officers due to ineffective screening tools, lack of knowledge of the local treatment landscape, ineffective referral practices (Knight et al., 2019), and a lack of collaboration between CS and BH agencies (Chuang & Wells, 2010; Taxman & Belenko, 2011). Consequently, CS agencies often develop internal solutions to address SU, such as the co-location of services (e.g., BH providers housed at juvenile probation departments). Co-location can facilitate integrated service delivery by primary care providers and BH specialists (Ragunanthan et al., 2017; Williams et al., 2006). Several studies have also noted that inter-organizational collaboration between CS and BH systems improved service access (Bai et al., 2009; Cottrell et al., 2000).

### Rationale for the current study

This study focuses solely on youth under CS (not institutionalized) in an effort to delineate the availability and quality of SU treatment services for JJ youth receiving services while living in their home setting. More than a decade has passed since a national survey of SU treatment service availability was conducted (Henderson et al., 2007; Young et al., 2007), and a more current snapshot is needed, particularly among youth committed to probation by the juvenile court. Therefore, the current study uses data from 2015 to augment prior studies and expands data collection to include perspectives from both CS and BH service providers.

This study addresses three questions: (RQ1) What treatment modalities are available to CS youth nationally? (RQ2) Do community treatment agencies serving CS youth utilize EBPs? (RQ3) Is collaboration with providers associated with wider service modality options, higher EBP utilization, or higher proportions of CS youth served? Co-location of services was expected to be associated with interagency collaboration, availability of treatment, and proportion of youth in need of care receiving services. Availability of treatment was expected to influence EBP utilization. Finally, both the availability of treatment and EBP utilization were expected to influence the proportion of youth served. Given the richness of data from both CS and BH agency perspectives, the current study offers a unique examination of collaboration and its potential impact on the availability and quality of SU treatment services.

## Methods

Data were collected between April 2014 and March 2015 as part of the NIDA-funded Juvenile Justice Translational Research on Interventions for Adolescents in the Legal System (JJ-TRIALS) cooperative and are a nationally representative sample of CS agencies (i.e., probation) and their primary BH providers. The methods (including respondent selection procedures) and main survey findings have been reported elsewhere (Scott et al., 2019).

### Sampling, recruitment, and weighted estimates

All agencies serving youth on CS across the 192 sampled counties were identified and surveyed regardless of the number of youth served. As outlined in Scott et al. (2019), counties were selected using a three-stage national probability sampling process that included states, counties, and CS agencies within counties. States and counties were stratified by the number of youth aged 10 to 19 residing in them, as documented in the 2010 Current Population Survey (CPS; U.S Census, 2012). The five largest states were selected with certainty. The remaining 15 were selected with probabilities proportionate to the number of youth in five population strata to ensure that less-populated states were included in the study. Within each state, the largest county and any other mega-counties (with 250,000 or more youth or half or more of the state’s youth in smaller states) were selected with certainty. The remaining counties were selected with probabilities proportionate to the number of youth in those counties. In the two small sampled states organized by judicial district instead of counties, all districts were sampled. Of the 192 sampled counties, only 10 had multiple CS agencies (9 had two, and 1 had three). Surveys were completed by 195 of 203 (96%) CS agencies. Data were weighted based on the inverse of the inclusion probability and were adjusted for nonresponses within states. The number of agencies overall and those providing a specific service were estimated by multiplying the weighted average number of agencies per county by the number of counties (*n* = 3,143). For youth characteristics, the weight was further adjusted to account for number of youth served so that the estimate better represented all youth on CS (*N* = 770,323).

Within each county, CS agencies were asked to identify the BH providers that served the most youth under CS. A total of 283 BH providers were identified and 271 (96%) were completed and returned. The total number of BH providers was estimated based on the weighted average number of BH providers per county multiplied by the number of counties (*n =* 3,143 counties). The number of BH agencies providing each specific service was estimated by multiplying the weighted average number of agencies times the number of BH agencies (*n* = 4,252 BH providers). For youth characteristics, the provider weight was multiplied by the number of youth served to represent the estimated number (*n* = 548,613) of youth on CS served by at least one primary BH provider.

BH provider data were merged with CS agency data at the CS agency level. When there were multiple CS agencies per county, the identified BH providers were matched with their corresponding agencies. In cases where SU and MH treatment were primarily delivered by two service providers (*n* = 86), data were aggregated into one BH provider record. For dichotomous items (0/1 for no/yes), the max (1/yes) across BH providers was used to create the matching BH provider variable for that CS agency’s record. For continuous items, the average across BH agencies was used. Aggregating the BH provider data resulted in one record per CS agency (192 records, unweighted; 3,202 weighted estimates).

### Measures

*Availability of Treatment Modalities* was calculated separately for CS and matching BH providers and included the count of outpatient, intensive outpatient, residential, co-occurring disorder, continuing/aftercare, and medication-assisted treatment (MAT) modalities reported within the county during the past year (Table 1). *Evidence-based Practice (EBP) Utilization* was assessed using four measures. For *Any Use of EBPs* and *Use of EBP with 50% or More Youth*, respondents were given a list of 29 EBPs (e.g., National Registry of Evidence-Based Practices and Programs, Crime Solutions; see Scott et al., 2019) and asked to indicate (during the past year) whether each was available at their agency (e.g., Motivational Enhancement Therapy) and to report the percentage of youth that received one or more EBP (Table 2). For *Minimum Education*, respondents indicated the minimum counselor education requirements for each service modality [minimum ≥ Master’s degree (MA, MS, MSW) requirement = 1; < Master’s degree = 0]. For *Family Involvement*, any practice that included family members was coded as 1 (e.g., family counseling, Brief Strategic Family Therapy, Functional Family Therapy, Multidimensional Family Therapy).

**Table 1:** Available Behavioral Health Service Modalities by Type of Provider

Service	CS Directly %	BH Directly %	Other External %	Not Available/ Not Know %
Outpatient Services	9	93	6	1
Among Outpatient:				
% Programs requiring Master’s degree	14	56	na	na
% Programs offering group	46	87	na	na
% Programs offering individual	68	96	na	na
% Programs offering family	61	80	na	na
% Programs offering telephone	7	35	na	na
% Total youth served^a,b,c^	10	38	na	na
Co-occurring Substance Use and Mental Health Treatment	5	80	17	3
Among SU and MH Treatment:				
% Programs requiring Master’s degree	8	78	na	na
% Total youth served	3	13	na	na
Continuing or Aftercare	4	68	25	10
Among Continuing/Aftercare:				
% Programs requiring Master’s degree	20	52	na	na
% Total youth served	2	11	na	na
Intensive Outpatient	1	39	48	15
Among Intensive Outpatient:				
% Programs requiring Master’s degree	11	60	na	na
% Total youth served	3	6	na	na
Other Recovery Support	1	25	55	21
Among Other Recovery Support:				
% Programs requiring Master’s degree	0^d^	17	na	na
% Total youth served	7	10	na	na
Residential Treatment	1	10	65	25
Among Residential:				
% Programs requiring Master’s degree	11	22	na	na
% Total youth served	5	8	na	na
Medication-Assisted Treatment (MAT)	---	7	62	31
Among MAT:				
% Programs requiring Medical degree	---	74	na	na
% Programs with a physician prescribing and/or managing meds	---	32	na	na
% Programs with onsite medication management	---	13	na	na
% Programs referring to a physician for a prescription and/or management	1	36	na	na
% Total youth served	---	---	na	na
Detoxification	---	4	57	39
Among Detoxification:				
% Programs requiring Master’s degree	---	62	na	na
% Total youth served	---	3	na	na

^a^Mean and % youth served based on agencies directly providing that service

^b^Annually

^c^Total youth based on youth served by the CS agency

^d^100% required a Bachelor’s degree

-- indicates the number is too small to estimate reliably

na indicates not asked since the service was not provided directly

*Notes*: First two columns are not mutually exclusive. “Other External” = neither CS nor BH directly provide the service, but one or both report it is available at an external agency within the county. Not Available/Not Know = both CS and BH report the service is not available in their county or that they do not know. CS = Community Supervision; BH = Behavioral Health

Data are weighted to reflect the estimated national population estimate of the 4,252 primary BH service providers and 3,202 CS agencies in the U.S. between April 2014 and March 2015 and have been adjusted for survey non-response at the state level

**Table 2:** Percentages of Programs using Evidence-Based Practices

EBP Interventions	Service System (Total) %	Community Supervision (CS) %	Behavioral Health (BH) %	
One or more EBP interventions indicated	86	10	88	
Any Motivational Enhancement (i.e., ME, MET, Marijuana Checkup)	76	10	75	
Any Cognitive-Behavioral (i.e., CBT w/o MET, MET/CBT, Seeking Safety and Teen Intervene)	70	3	73	
Any Family	46	3	45	
(i.e., FBT, BSFT, FSN, MDFT, FFT, Family Matters, MST and PLL)				
Any Individualized Approaches	19	3	17	
(i.e., Contingency Management/ Motivational Incentives and A-CRA)				
Any Other Substance Use Approaches	13	4	11	
(i.e., 7 C, Phoenix House Academy, ACC, CSH-OP and Behavior Management through Adventure)				

*Note.* Percentages are based on agencies/providers that reported using at least one of the interventions in the grouping. The sum is the count of the 5 categories within which the agency/provider reports using EBP interventions. EBP = Evidence-Based Practice; MI = Motivational Interviewing; MET = Motivational Enhancement Therapy; CBT = Cognitive-Behavior Therapy; FBT = Family Behavior Therapy; BSFT = Brief Strategic Family Therapy; FSN = Family Support Network; MDFT = Multidimensional Family Therapy; FFT = Functional Family Therapy; MST = Multisystemic Therapy; PLL = Parenting with Love and Limits; A-CRA = Adolescent Community Reinforcement Approach; 7 C = Seven Challenges; ACC = Assertive Continuing Care; CSH-OP = Chestnut Health Systems Outpatient

*Co-location of Services* was coded “yes” (1), if the CS agency reported providing office space for BH services or if one or more SU treatment modalities were provided directly by the CS agency. *Interagency Collaboration* was assessed by asking respondents about their working relationship with the other agency (CS-rated BH and BH-rated CS; Table 3); items were summed to form an index. *Proportion of Youth Served* was calculated by dividing the total youth receiving SU services by the total youth on CS. If only one agency reported a direct service, that number was used; if both CS and BH reported a direct service, the number of youth served was summed.

**Table 3:** Inter-agency Collaboration across the Service System

					Across Service System
	CS	BH	Neither CS nor BH	Either CS or BH	Both CS and BH
Activities	%	%	%	%	%
1. Hold joint staffings	85	67	8	37	55
2. Written protocols for sharing information	56	74	12	52	36
3. Have agreed to similar requirements for program eligibility	67	46	23	44	33
4. Modified protocols to meet the needs of this partner agency	29	53	36	49	15
5. Cross-train staff with this partner	31	17	65	24	11
6. Pooled funding to provide services	19	18	73	19	8
7. Shared operational oversight of treatment programs	18	17	75	17	8
8. Shared budgetary oversight of treatment programs	11	13	82	14	5
9. Developed joint policy/procedure manuals	13	12	75	23	0.3
Collaboration Index (“yes” on 1 + items 1–9)	96	87	--	--	64

*Note*: CS = Community Supervision; BH = Behavioral Health

Respondents were asked to report the number of non-clinical, clinical, and medical FTEs employed to serve youth on CS. Clinical FTEs were dichotomized into any versus no clinical FTEs. The percentages of youth from minority races (including BIPOC and Hispanic ethnicities) served were summed to derive the percentage of the youth caseload that was of minority status. Survey respondents were asked to report percentages of youth on their caseload with any SU problems (including alcohol), alcohol problems, marijuana problems, and prescription drug problems. The maximum percentage reported across items was used to measure the percentage of youth served with SU problems.

### Analysis

Descriptive statistics addressed RQ1 and RQ2. Structural Equation Modeling (SEM; using IBM SPSS AMOS version 25) was used to model the relations among interagency collaboration, modality options, best practice utilization, and proportion of youth served (RQ3). After examination of the hypothesized model, paths with an alpha ≥ 0.05 were deleted. New paths were checked at each step, and all exogenous variables were allowed to co-vary. For each step, model fit was examined using the minimum fit chi-square, the comparative fit index (CFI), and the root mean error of approximation (RMSEA). RMSEA values < 0.05 indicate good fit, 0.08 or less indicate moderate fit, and values > 0.10 indicate poor fit. CFI ranges from 0 to 1, with values > 0.95 indicating very good fit. To control for agency and youth variance, a second model was estimated. The addition of covariates had no effect on the existing path coefficients and resulted in a poorer model fit. Therefore, only the model without covariates is presented. The data were weighted to represent the national population of the 3,202 CS agencies.

Missing data were less than 1% for all variables except for the proportion of youth served, which was missing for 11% of the unweighted records, particularly for smaller and rural counties with large weights. Maximum likelihood estimation was used in AMOS to account for missing data.

## Results

Service systems were located in urban (39%), rural, (37%), and urban-adjacent (24%) settings. Most (66%) were operated by the respective state (32% county). The majority of youth served were male (73%), aged 14–15 (41%) or 16–17 (40%), and White (75%; 12% Black, 8% Hispanic/Latino, 5% Other). All systems provided post-adjudication supervision, 85% detention, 79% pre-adjudication supervision, 68% long-term post-adjudication residential, and 23% assessment. On average, systems reported SU problems among 49% of youth, with the most problematic substance being marijuana (39%) followed by alcohol (27%), prescription drugs (16%), and other (12%; amphetamine/methamphetamine, cocaine/crack, heroin, etc.). 29% of systems reported co-location of CS and SU services.

As expected (RQ1), a higher proportion of youth on CS received services through BH rather than CS (see Table 1). The most common SU treatment modality was outpatient (93% of BH, 9% of CS provided directly). But, while nearly all BH agencies reported offering outpatient services, a small proportion of CS youth received them (38% at BH, 10% at CS). Co-occurring SU and MH services were most often offered by BH (80%) and other external agencies (17%), with only 5% of CS providing co-occurring services directly; thus, relatively few CS youth received these services. While continuing/aftercare services were offered by most BH agencies (68%); few CS agencies provided continuing/aftercare (4%) and 10% indicated they did not know of any agencies in the county offering aftercare services for youth on CS. Other types of available services included intensive outpatient (48%), other recovery support (55%), residential (65%), MAT (62%), and detoxification (57%). A sizeable number of BH and CS respondents did not know where to access residential (25%), MAT (31%), and Detox (39%) in their county.

In general, BH providers reported greater use of EBPs compared to CS agencies; 88% of BH reported one or more compared to 10% CS (see Table 2; RQ2). The most common EBPs used were motivational enhancement (75% BH, 10% CS) and cognitive-behavioral strategies (73% BH, 3% CS). Less than half of BH (45%) and only 3% of CS agencies reported any family involvement. Few used individualized or other SU approaches. Across the system, 59% reported using an EBP with 50% or more youth, and 36% reported a minimum counselor education requirement of an MS degree for staff.

Both CS and BH agencies reported holding joint staffing meetings (55%), developing written protocols for sharing information (36%), and agreeing to similar program requirements (33%; see Table 3). Most did not share budgetary (82%) or operational oversite (75%), develop a joint policy/procedure manual (76%), or pool funding (73%). In terms of discrepancies, CS reported more joint meetings (85% vs. 67% BH) and agreeing to similar program requirements (67% vs. 46% BH); however, BH reported more written protocols for sharing information (74% vs. 56% CS) and more protocol modifications to meet the needs of the partner agency (53% vs. 29% CS).

Means, standard deviations, and zero-order correlations (see Table 4) indicate significant yet modest relations between the *Co-location of Services* with *Interagency Collaboration* (*r* = .25) and *Availability of Treatment Modalities* (*r* = − .09). The four items measuring *EBP Utilization* (*Any EBP, EBP with 50% or More Youth, Family Involvement, Minimum Education*) were related to each other with correlations ranging from 0.11 to 0.28. The exception was no relation between *Family Involvement* and *EBP with 50% or More Youth* (*r* = .00). *Availability of Treatment Modalities* and *EBP Utilization* were related to *Proportion of Youth Served*, with correlations ranging from 0.22 to 0.30.

**Table 4:** (Appendix) *Correlations, Means, and Standard Deviations for Key Variables*

	1	2	3	4	5	6	7	M	SD
1. Co-location	--	--	--	--	--	--	--	0.29	0.45
2. Interagency Collaboration	0.25*	--	--	--	--	--	--	1.72	1.98
3. Availability of Treatment Modalities	− 0.09*	0.02	--	--	--	--	--	2.93	1.18
4. Use of Any EBPs	0.00	− 0.04*	0.14*	--	--	--	--	0.86	0.35
5. Use EBP with 50%+ Youth	− 0.05*	0.18*	0.26*	0.16*	--	--	--	0.59	0.49
6. Any Family Involvement	− 0.12*	0.14*	0.22*	0.24*	0.00	--	--	0.80	0.40
7. Minimum MS Education	− 0.04*	0.06*	0.02	0.12*	0.28*	0.11*	--	0.36	0.48
8. Proportion of Youth Served	0.00	− 0.17*	0.27*	0.27*	0.30*	0.29*	0.22*	0.48	0.40

*Note.* * *p* < .05

**Fig. 1:**
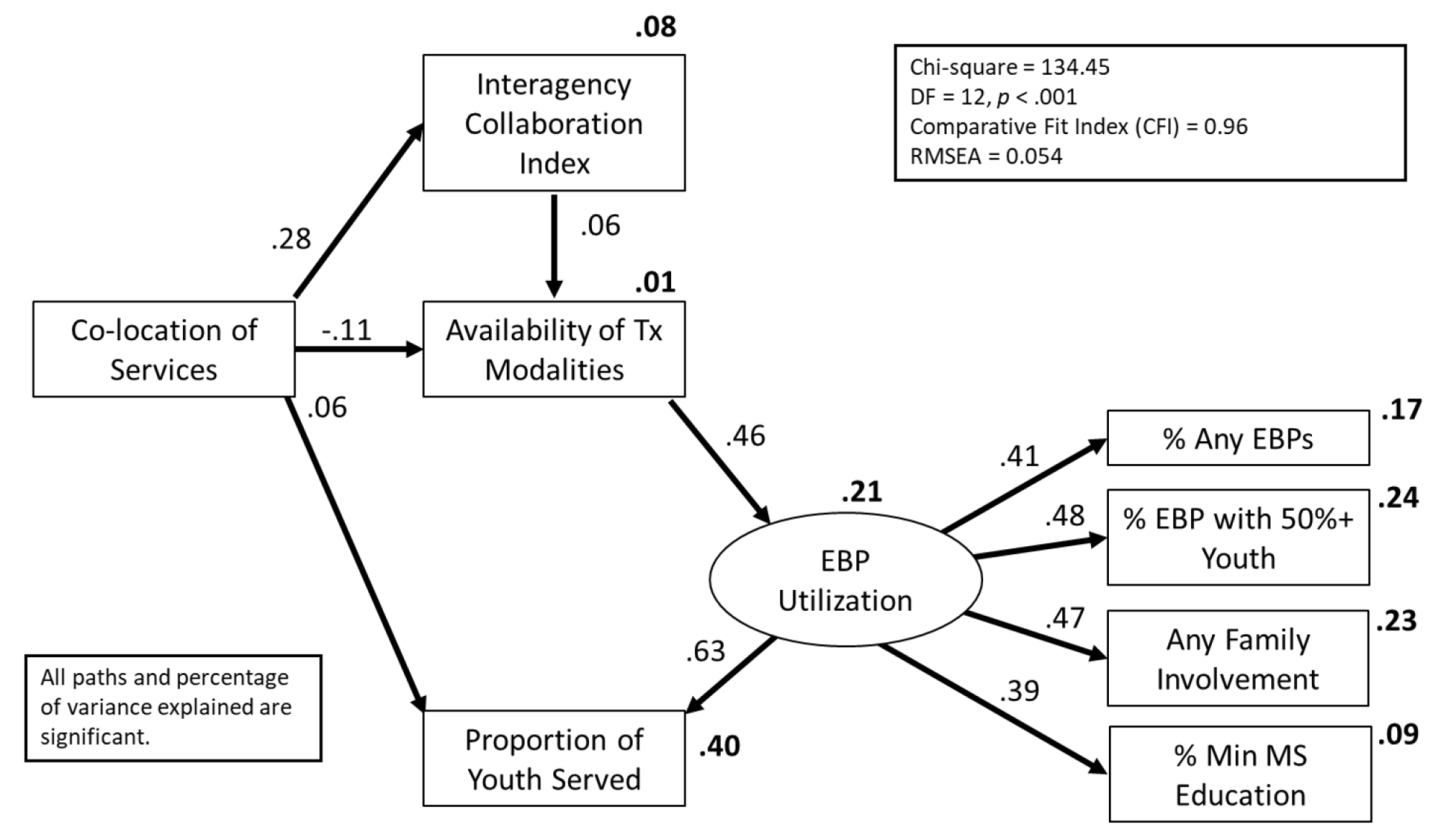
Final Structural Equation Model for Interagency Collaboration, Modality Options, EBP Utilization, and Proportion of Youth Served Note. EBP = Evidence-Based Practice; RMSEA = Root Mean Square Error of Approximation

Figure 1 shows the final model in which the paths with standardized coefficients not significantly different from zero were omitted (RQ3). The final model showed good fit (χ^2^ [12] = 134.45, *p* < .001) with a CFI = 0.96 and a RMSEA = 0.054. While the chi square test was significant, CFI and RMSEA indicate good to moderate fit, and 8 of 10 coefficients were 0.28 or higher. No other individual paths were significant or further improved fit. Contrary to expectations, there was a significant direct path between *Co-location* and the *Proportion of Youth Served* (β = 0.06; note, however, the small coefficient). Furthermore, *EBP Utilization* appeared to mediate the path between *Availability of Treatment Modalities* and *Proportion of Youth Served* (*Avail of Tx* to *EBP Utilization*, β = 0.46; *EBP Utilization* to *Prop Youth*, β = 0.63). Without *EBP Utilization* in the model, the path from *Availability of Treatment Modalities* to *Proportion of Youth Served* was significant (β = 0.27).

## Discussion

This study documents SU treatment services available for a national sample of youth under CS. The most common SU treatment modality was outpatient; however, services were only available to less than half of youth (RQ1). Across modalities, youth on CS were most likely to receive services through BH or other external agencies. Many respondents did not know where to access county-level residential, MAT, and detoxification services.

The results indicated gaps in EBP use (RQ2). BH providers were more likely to use EBPs compared to CS, especially motivational enhancement and cognitive-behavioral approaches. Across BH and CS agencies, less than half reported family involvement in SU service provision. Similarly, few used individualized (e.g., contingency management) or other SU approaches (e.g., Assertive Continuing Care).

Structural equation modeling was employed to analyze RQ3. In the final model, interagency collaboration appears important, as it was significantly associated with a higher number of treatment modalities available, despite the low beta coefficient. The degree of interagency collaboration among BH and CS agencies varied and included joint meetings, written information-sharing protocols, and coordinated program requirements. In turn, more treatment modalities were related to higher EBP utilization, which was related to a higher proportion of youth served. When BH services are largely available but CS agencies are under-utilizing or are unaware of local providers, CS agencies should improve connections to existing networks and build collaborations in their community. Fruitful avenues may include the development of interagency work teams designed to address local service gaps, develop shared referral protocols, and share service attendance information as part of contractual agreements (Knight et al., 2016; Welsh et al., 2021).

The BH Services Cascade illustrates the interrelated series of events experienced by CS youth as the system attempts to identify SU needs and link them to treatment (Belenko et al., 2017). Implementing universal evidence-based screening protocols upon entry into the JJ system would increase the likelihood that every youth with a SU need would be identified (Wiese et al., 2019). While passive referrals (e.g., family is encouraged to make an appointment) are common among CS staff (Knight et al., 2019), directed referrals (e.g., CS staff arranging an initial appointment at a partner agency, providing transportation) increase treatment initiation (Clemens et al., 2006; Rastegar, 2012). These “warm handoffs” promote a smooth transition to treatment and recovery (Miller-Matero et al., 2016). Because youth rely on caregivers for logistical and emotional support to overcome SU issues, more work is needed to increase family engagement when a SU referral is made so that existing support networks can be leveraged.

Barriers to treatment access also include difficulties in navigating CS and BH systems. When housed in different locations, families must travel to receive services, rely on others when transportation is unavailable, and complete extensive paperwork before initiating services. People of color often disproportionately encounter additional barriers, such as living in “service deserts” where few or no providers exist in their geographic area (Sager, 2013). Regardless of availability and access, there may be few providers willing to work with youth on CS.

Several limitations should be acknowledged. Although the potential for reporting bias exists, response rates were high (96%). Respondents likely represented different positions within the agencies, as well as different backgrounds, knowledge, training, and lengths of experiences. This flexible approach was intentional, due to structural diversity among agencies and a desire to secure the most valid data possible from those with accurate knowledge. Second, many programs did not collect information on specific BH needs of the youth receiving services. Relatedly, record-keeping practices differed, even within state data systems; hence, the availability of detailed information regarding practices was limited for some sites. Inconsistencies in terminology, measures, and definitions across CS and BH agencies presented additional challenges to interpretation. While it would have been helpful to verify through observation that, for example, specific EBPs were indeed offered, the resources required to do so were not available for the current study. Clearly, some agencies exhibit higher quality data than others, and a separate study could be undertaken to categorize various types of data enhancement opportunities. It is also worth noting that data reported in the current study were collected at a single point in time between 2014 and 2015, and therefore, causality cannot be inferred. Investigations exploring similar research questions are an important next step in understanding facilitators and barriers to treatment for JJ youth. For example, during the COVID-19 pandemic, JJ agencies were forced to make rapid adjustments to long-standing policies and practices to abide by local public health measures, such as implementing telework policies for staff (Lockwood et al., 2023). The role of local funding priorities should also be considered, as these could account for the relationship between more modalities and higher EBP utilization. Future studies should also further explore the consequences of allowing the use of technology and telework, and whether they enhance or hinder service delivery for JJ youth. Finally, *Proportion of Youth Served* was based on aggregate estimates provided by agency respondents, and *Any EBP* was used as a gross proxy measure of service quality. Since individual youth records were not collected as part of this national survey, this study relied on agency-level records and therefore could not determine the number of youth served across multiple agencies (i.e., could not control for the chance that both CS and BH reported servicing the same youth). Additionally, limited study resources necessitated the combining of all surveyed SU and MH agencies in a given community into a single agency. Future studies should utilize youth records data to gain more reliable measures and potentially utilize alternative strategies for measuring EBP utilization. Despite these limitations, results provide insights into disparities that plague youth treatment access and highlight effective practices for increasing youth access to services.

## Conclusions

As expected, the co-location of SU services within the CS agency (whereby youth access treatment services and CS appointments in the same place) was associated with greater interagency collaboration and potentially a greater proportion of youth served but with fewer available treatment modalities. This pattern suggests that co-location may increase treatment availability when few options exist within the local community, however, the small coefficients indicate that future studies are warranted. When youth can access CS and BH appointments in one location, families may possibly overcome multiple logistical barriers due to easier access, familiarity with the facility, and efficiency in scheduling. Additionally, BH and CS staff can collaborate more closely, both formally and informally. For example, if a youth misses a BH appointment, BH staff can communicate directly with CS staff about attendance and needs and work together to address those needs.

While co-location may increase the likelihood of BH service receipt among CS youth, understanding the role of interagency collaboration requires further research. Tapping into existing networks to provide a broader array of treatment modalities appears to be beneficial for service access, but simply expanding existing service networks may not be sufficient to reach all youth. Efforts are underway to understand how improving collaboration can facilitate more appropriate and efficient progression through the BH Services Cascade when SU treatment is needed (Rastegar, 2012). The current findings identify opportunities to improve linkage to care by improving access, coordination, and use of EBPs to address the SU service needs of CS youth.

## Data Availability

The current protocol is part of the broader Juvenile Justice-Translational Research for Adolescents in the Justice System (JJ-TRIALS) Cooperative with a total of six research centers and one Coordinating Center. All data were de-identified prior to creation of the master dataset. At project close, data will be shared publicly, following NIDA guidance for appropriate public access and data sharing plans. Sharing of underlying primary data for publications will be made broadly available through an appropriate data repository.
